# Evaluation of combined testing to simultaneously diagnose pituitary pars intermedia dysfunction and insulin dysregulation in horses

**DOI:** 10.1111/jvim.15617

**Published:** 2019-09-09

**Authors:** Remona Horn, François‐René Bertin

**Affiliations:** ^1^ School of Veterinary Science University of Queensland Gatton Queensland Australia

**Keywords:** ACTH, diagnostic test, endocrinology, glucose, insulin

## Abstract

**Background:**

The thyrotropin‐releasing hormone (TRH) stimulation test and the 2‐step insulin sensitivity test are commonly used methods to diagnose, respectively, pituitary pars intermedia dysfunction (PPID) and insulin dysregulation (ID).

**Objectives:**

To investigate the diagnostic value of combining the TRH stimulation test and the 2‐step insulin sensitivity test to diagnose PPID and ID simultaneously.

**Animals:**

Twenty‐seven adult horses, 10 control horses without PPID or ID, 5 horses with PPID only, 5 horses with ID only, and 7 horses with PPID and ID.

**Methods:**

Randomized prospective study. Horses underwent a TRH stimulation test alone, a 2‐step insulin sensitivity test alone, and combined testing with simultaneous TRH and insulin injection in the same syringe. Data were compared by 2‐way repeated measures analysis of variance and 2 1‐sided tests to demonstrate equivalence. Bland‐Altman plots were generated to visualize agreement between combined and independent testing.

**Results:**

The effect of combined testing on plasma adrenocorticotropic hormone, blood glucose concentration, or percentage decrease in blood glucose concentration was not significantly different from the effect obtained with independent testing. One control horse appeared falsely positive for PPID, 2 PPID‐only horses appeared falsely positive for ID, and 1 PPID and ID horse appeared falsely negative for ID when tests were performed simultaneously. Bland‐Altman plots supported the agreement between combined and independent testing.

**Conclusions and Clinical Importance:**

Combining the TRH stimulation test and the 2‐step insulin sensitivity test appears to be a useful diagnostic tool for equine practitioners in the field, allowing testing of a horse for both PPID and ID simultaneously.

**Abbreviations**
ACTH: adrenocorticotropic hormone
EMS: equine metabolic syndrome
ID: insulin dysregulation
OST: oral sugar test
PPID: pituitary pars intermedia dysfunction
TOST: 2 1‐sided test
TRH: thyrotropin‐releasing hormone

## 1 INTRODUCTION

Pituitary pars intermedia dysfunction (PPID) and insulin dysregulation (ID) are the most common hormonal disorders in adult horses.^1^, ^2^, ^3^ Pituitary pars intermedia dysfunction is commonly detected in horses older than 15 years, with up to 30% of geriatric horses affected.^4^, ^5^, ^6^ This disease is caused by neurodegeneration of the inhibitory dopaminergic neurons of the hypothalamus resulting in hyperplasia of the pars intermedia of the pituitary gland. The hormone‐secreting hyperplastic tissue releases an unregulated amount adrenocorticotropic hormone (ACTH).^7^, ^8^ Measurement of baseline plasma ACTH concentration is the most frequently used method to diagnose PPID worldwide.^6^ However, the thyrotropin‐releasing hormone (TRH) stimulation test is a dynamic test that has been shown to be more sensitive for the diagnosis of PPID, especially in early stages of the disease when clinical signs are vague or when baseline ACTH concentrations are still within the reference range.^9^, ^10^ The TRH stimulation test is now a recommended test to diagnose early PPID (Equine Endocrinology Group website, available at: http://sites.tufts.edu/equinendogroup, accessed January 10, 2019). In addition to its increased sensitivity, this test can be performed within 30 minutes, and because TRH has been shown to be stable at room temperature, the test could be used in practice.^11^

Equine metabolic syndrome (EMS) is a complex disorder characterized by increased adiposity, a predisposition to laminitis, and multiple metabolic dysregulations.^12^ The disease process of EMS still is incompletely understood, but it is known that ID is a core component of this disorder and is associated with the development of laminitis.^13^, ^14^, ^15^ Insulin dysregulation encompasses tissue insulin resistance, persistent or intermittent hyperinsulinemia, or a combination of tissue insulin resistance and persistent or intermittent hyperinsulinemia.^13^ Several tests have been developed to diagnose ID, some assessing insulin response to a glucose challenge, some assessing glucose response to an insulin challenge, and some assessing both.^13^, ^16^, ^17^ The oral glucose test and oral sugar test (OST) are commonly used tests and are based on the PO administration of dextrose or corn syrup to fasted horses and measurement of the insulin response to determine the presence of hyperinsulinaemia.^6^, ^18^ These tests require an 8‐hour to 10‐hour fast as well as 2 hours to complete the test.^19^, ^20^ The 2‐step insulin sensitivity test has been shown to be a safe, sensitive, and repeatable test to diagnose horses with peripheral tissue insulin resistance and can be performed within 30 minutes without any fasting, indicating that the test could be easily used in practice.^16^

Subclinical PPID can be challenging to detect, especially when it develops in a horse with ID in which subtle clinical signs could be attributed to deterioration in insulin regulation.^5^ Similarly, in horses suffering from PPID, higher rates of therapeutic failure have been observed when ID is unrecognized.^6^ One previous study has investigated the combination of the TRH stimulation test and the OST.^21^ However, PO administration of glucose to horses significantly impacted their plasma ACTH concentrations after TRH injection.^21^ Therefore, our aim was to investigate the diagnostic value of the TRH stimulation test and the 2‐step insulin sensitivity test performed in combination to provide a diagnostic protocol for PPID and ID simultaneously. We hypothesize that measured concentrations obtained from the combination test would not differ from the concentrations obtained when the tests are performed independently.

## 2 MATERIALS AND METHODS

### 2.1 Horses

Twenty‐seven horses and ponies (15 gelding and 12 mares) from the institutional research herds were included in the study. The median age of the horses was 18 years (range 9‐27 years) and median weight was 492 kg (range 143‐650 kg). When a scale was not available on the premises, body weight was estimated using a weigh tape.^22^ Breeds included Australian Stock Horse (n = 11), Standardbred (n = 6), mixed Ponies (n = 6), Warmblood (n = 2), and Arab (n = 1). None of the horses included in the study had been diagnosed previously with PPID or ID and none had received any treatment. However, 4 horses had clinical signs suggestive of EMS such as regional adiposity and hoof rings, 3 horses had clinical signs suggestive of PPID such as hypertrichosis and delayed shedding, and 6 horses had clinical signs suggestive of both disorders. All horses were considered healthy based on physical examination findings. The study protocol was approved by the Institutional Animal Ethics Committee.

### 2.2 Study design

To test the characteristics of combined testing compared to independent testing, 4 groups of horses were used. The first group of horses included horses with neither PPID nor ID (control group). The second group included horses with ID but no PPID (ID‐only group). The third group included horses with PPID but no ID (PPID‐only group). The fourth group included horses with both PPID and ID (PPID and ID group). All horses underwent the same series of tests in a randomized order: a TRH stimulation test alone, a 2‐step insulin sensitivity test alone, and combined testing (a 2‐step insulin sensitivity test and a TRH stimulation test). Some horses received the combined testing first, whereas others received 1 of the independent tests first.^9^, ^10^, ^16^ All tests were performed >24 hours apart but <2 weeks apart.^21^ The diagnoses of PPID and ID were based on results of the independent testing (TRH stimulation alone and 2‐step insulin response test alone). Water and food were available ad libitum before and after all tests. All horses were acclimatized to their environment and received the same farm care (institutional pastures). The tests were performed between October and February (spring and summer months).

### 2.3 Thyrotropin‐releasing stimulation test

Thyrotropin‐releasing hormone solution was prepared as a single batch. Fifty milligrams of synthetic TRH acetate salt (Sigma‐Aldrich Co, Castle Hill, Australia) was reconstituted using a sterile technique in a biological safety cabinet. Sterile water for injection was used to create a solution containing 1 mg of TRH/mL. One‐milligram aliquots were stored in sterile 3‐mL syringes at –20°C until the time of use. A jugular venipuncture was performed to obtain a 5‐mL blood sample immediately before to TRH treatment (baseline sample), and the sample was transferred to chilled tubes containing EDTA as an anticoagulant. One milligram of TRH then was administered into a jugular vein. The horses were observed for any adverse effects associated with the TRH administration during the following 30 minutes. A 5‐mL poststimulation blood sample was obtained by venipuncture 30 minutes after TRH administration. Blood samples were placed in a cooler with ice packs and centrifuged within 4 hours after collection. Plasma ACTH concentrations were measured by the institutional laboratory using a chemiluminescent immunoassay (IMMULITE 1000 Immunoassay System, Siemens Healthcare Pty Ltd, Bayswater, Australia) with an intra‐assay variability of 4.8%.^23^ The immunoassay used to measure plasma ACTH concentrations previously was validated for use in horses and the laboratory has conducted its own validation and quality control procedures.^23^ Results of the TRH stimulation test were considered positive if the baseline plasma ACTH concentration was >35 pg/mL or post‐stimulation plasma ACTH concentration was >65 pg/mL as described in the Equine Endocrinology Group recommendations (Equine Endocrinology Group website, available at: http://sites.tufts.edu/equinendogroup, accessed January 10, 2019).

### 2.4 Two‐step insulin sensitivity test

Regular human recombinant insulin (Actrapid R 0.1 IU/kg, Novo Nordisk, Auckland, New Zealand; 0.1 mIU/mL) was rapidly injected by jugular venipuncture, and blood samples were collected at time 0 (just before insulin injection) and 30 minutes after insulin injection. A hand‐held strip glucometer (AlphaTRAK2, Zoetis Inc, Kalamazoo, Michigan) with intra‐assay variability of 5.3% was used to determine all blood glucose concentrations. This device has been used previously in horses to diagnose peripheral tissue insulin resistance.^2^, ^24^, ^25^ To prevent potential hypoglycemia, 150 mg/kg 50% dextrose was administered IV after the 2‐step insulin sensitivity test was completed if blood glucose concentration was <1.1 mmol/L or if the horse showed clinical signs of hypoglycemia such as sweating, muscle fasciculations, or ataxia. The 2 blood glucose concentrations (baseline and 30 minutes after insulin injection) were used to determine peripheral tissue insulin sensitivity. A diagnosis of ID was made if the blood glucose concentration of the 30‐minute sample was higher than half the baseline concentration (less than a 50% decrease).^16^

### 2.5 Combined testing

All horses underwent combined endocrine testing. The TRH stimulation test and 2‐step insulin sensitivity test were performed as previously described with TRH (1 mg) and insulin (0.1 IU/kg) administered IV in the same syringe by jugular venipuncture. The baseline sample was used for the measurement of plasma ACTH concentration for the TRH stimulation test as well as the baseline blood glucose concentration. After injections, plasma ACTH and blood glucose concentrations were determined at 30 minutes as described above.

### 2.6 Statistical analysis

Binary outcomes (positive or negative for PPID and positive or negative for ID) were compared between independent and combined testing to assess the accuracy of the combined testing. Variables (plasma ACTH concentration, blood glucose concentration, and percentage decrease in blood glucose concentration) were assessed for normality by a Shapiro‐Wilk test. A paired *t* test (normally distributed data) or a Wilcoxon signed rank test (non‐normally distributed data) was used to compare baseline and post‐procedure plasma ACTH and blood glucose concentrations as well as percentage decrease in blood glucose between tests performed as independent and combined testing. Baseline plasma ACTH and blood glucose concentrations were compared between groups using a Kruskal‐Wallis test. A 2‐way repeated measure analysis of variance then was used to analyze the effect of combined versus independent testing as well as the effect of insulin and TRH administration on blood glucose and plasma ACTH concentrations, respectively. Equivalence of testing protocols was assessed using a 2 1‐sided test with the intra‐assay variability for ACTH and glucose assays to be compared to the observed difference. Bland‐Altman plots were determined to allow numerical assessment of the discrepancy between methods (bias) and variability in bias across the range of measured concentrations. All calculations were performed using commercial statistical software (Prism, GraphPad Software, Inc, La Jolla, California), and *P* values <.05 were considered significant.

## 3 RESULTS

**Figure 1:**
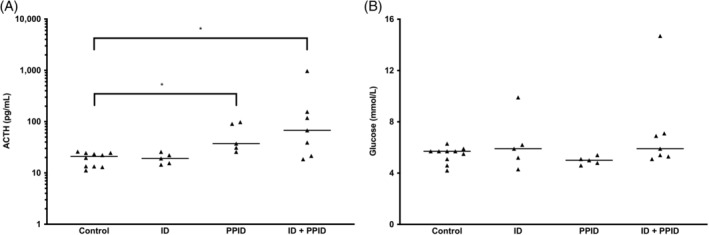
Plasma ACTH concentrations (A) and blood glucose concentrations (B) for the 4 groups of horses at baseline (* *P* < .05, between groups). ACTH, adrenocorticotropic hormone

Based on independent testing, the control group included 10 horses, the ID‐only group included 5 horses, the PPID‐only group included 5 horses, and the PPID and ID group included 7 horses. Baseline plasma ACTH concentrations differed significantly between groups with PPID (groups 3 and 4) and groups without PPID (groups 1 and 2; *P* = .01; Figure 1A). Baseline blood glucose concentrations did not differ significantly among any of the 4 groups (*P* = .10; Figure 1B).

All horses tolerated both testing protocols well and dextrose was only administered IV to 1 horse in the PPID group because of mild signs of hypoglycemia (sweating at 40 minutes with a blood glucose concentration of 1.2 mmol/L). No complications were observed after dextrose administration to the mare.

### 3.1 Diagnosis of PPID

**Figure 2:**
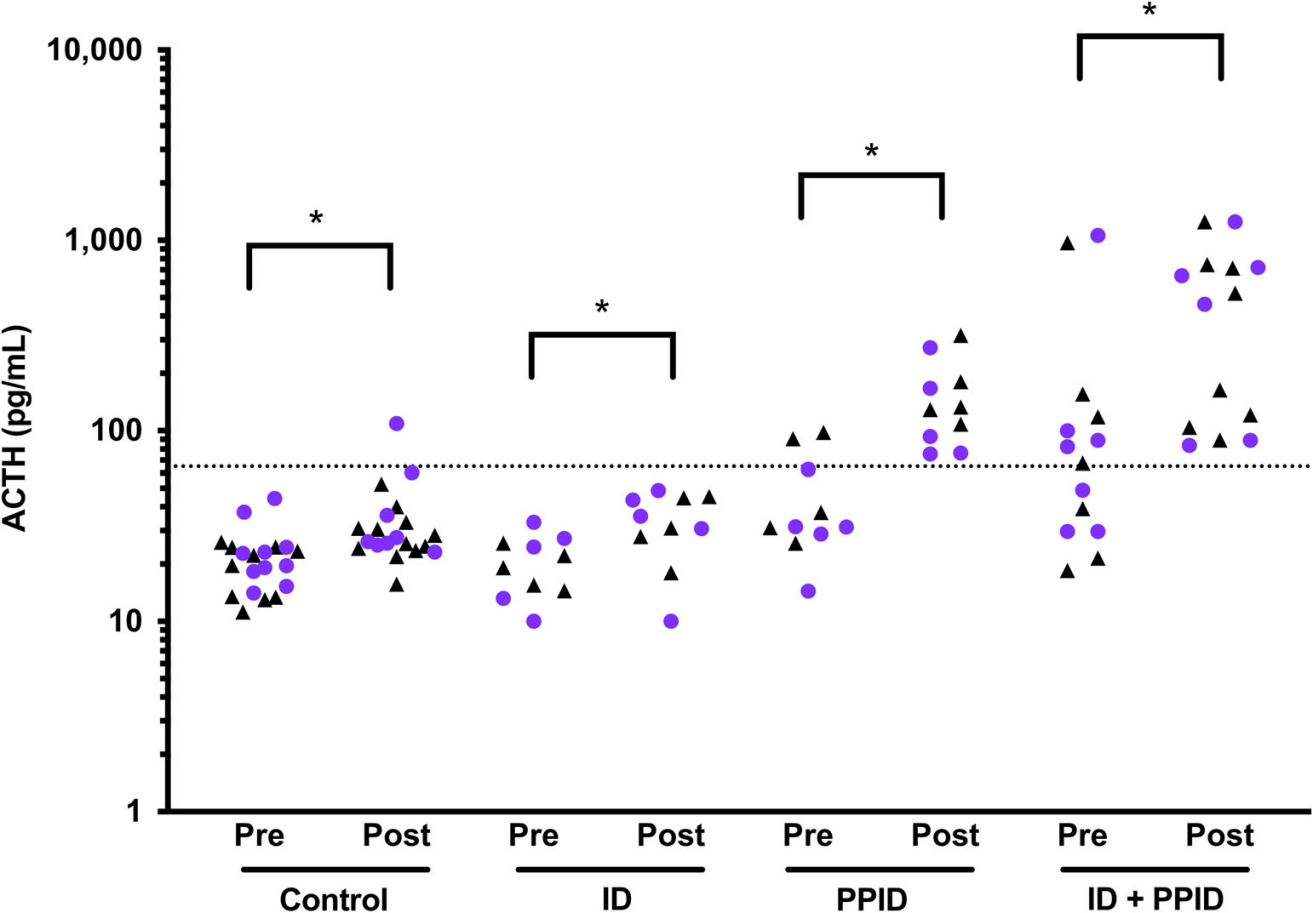
Plasma ACTH concentrations at baseline (pre) and after a TRH stimulation test (post) in the 4 groups of horses. Black triangles represent data from independent testing and purple circles represent data from combined testing (* *P* < .05 between pre‐stimulation and post‐stimulation). ACTH, adrenocorticotropic hormone

In the control group, no significant difference was noted in baseline plasma ACTH concentrations (*P* = .23) or in post‐stimulation plasma ACTH concentrations (*P* = .70) between combined and independent testing. The TRH administration had a significant effect on plasma ACTH concentration in both independent and combined testing (*P* = .01), but no significant effect of combining the 2 tests was observed (*P* = .26; Figure 2). One horse had a false‐positive TRH stimulation test result on the basis of a post‐stimulation plasma ACTH concentration of 109 pg/mL after 30 minutes with the combined testing.

In the ID‐only group, no significant difference was noted in the baseline plasma ACTH concentrations (*P* = .54) or in the post‐stimulation plasma ACTH concentrations (*P* = .97) between combined and independent testing. The TRH administration had a significant effect on plasma ACTH concentration in both independent and combined testing (*P* = .01), but combining the 2 tests did not have a significant effect (*P* = .82; Figure 2).

In the PPID‐only group, no significant difference was noted in the baseline plasma ACTH concentrations (*P* = .14) or in the post‐stimulation plasma ACTH concentrations (*P* = .18) between combined and independent testing. The TRH administration had a significant effect on plasma ACTH concentration (*P* = .02), but no significant effect of combining the 2 tests (*P* = .10; Figure 2).

In the ID and PPID group, no significant difference was noted in baseline plasma ACTH concentrations (*P* = .94) or in the post‐stimulation plasma ACTH concentrations (*P* = .59) between combined and independent testing. The TRH administration had a significant effect on plasma ACTH concentration (*P* = .02), but no significant effect of combining the 2 tests was observed (*P* = .36; Figure 2).

### 3.2 Diagnosis of equine ID

**Figure 3:**
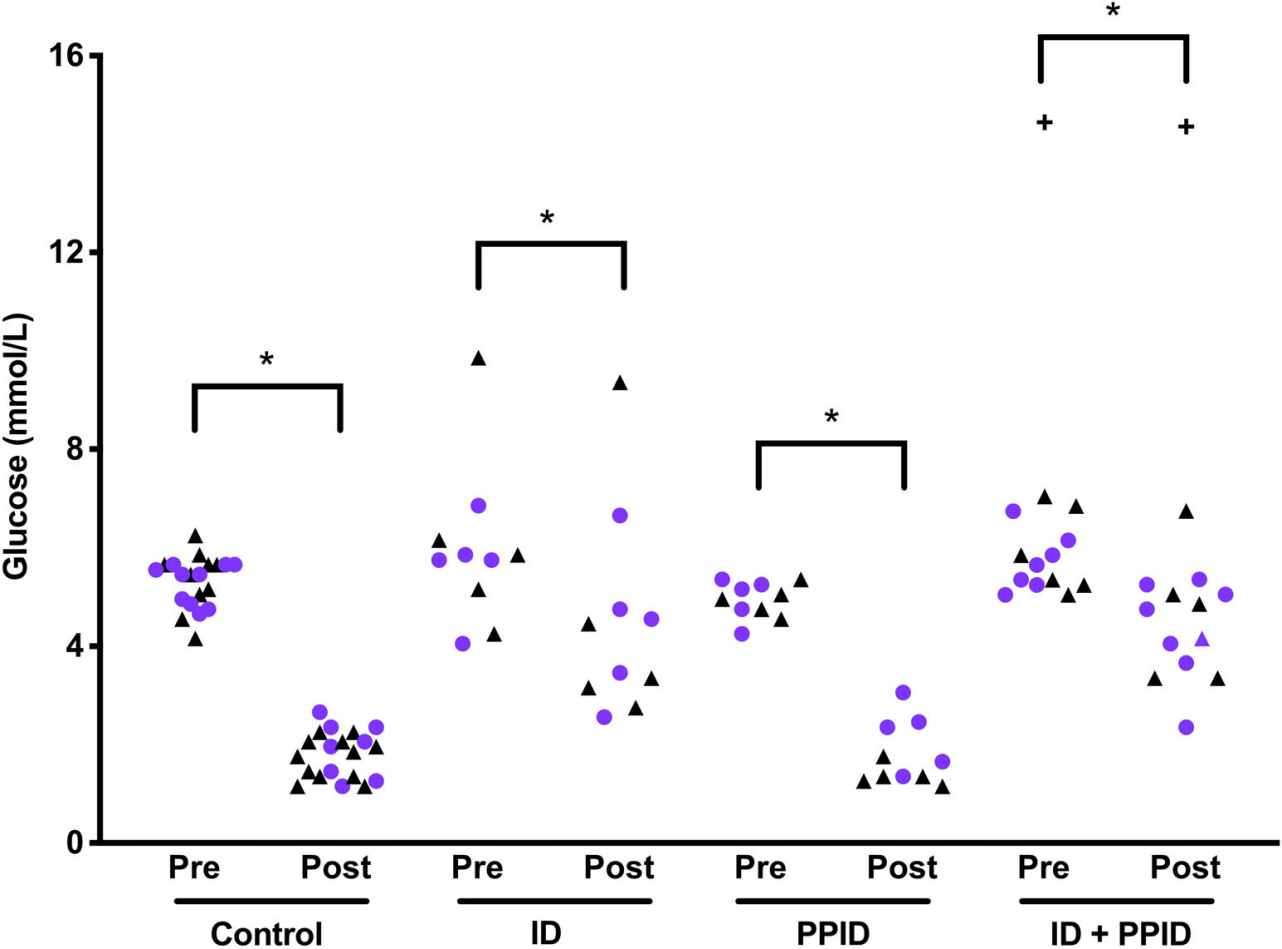
Blood glucose concentrations at baseline (pre) and after a 2‐step insulin sensitivity test (post) in the 4 groups of horses. Black triangles represent data from independent testing and purple circles represent data from combined testing (* *P* < .05 between pre‐stimulation and post‐stimulation)

In the control group, no significant difference was noted in baseline blood glucose concentrations (*P* = .43) or in post‐insulin blood glucose concentrations (*P* = .22) between combined and independent testing, and insulin administration had a significant effect on blood glucose concentration (*P* = .01). Combining the 2 tests did not have a significant effect (*P* = .89, Figure 3).

In the ID‐only group, no significant difference was noted in baseline blood glucose concentrations (*P* = .55) or in post‐insulin blood glucose concentrations (*P* = .81) between the independent and combined testing. Insulin administration had a significant effect on blood glucose concentration in both independent and combined testing (*P* = .01). No significant effect of combining the 2 tests was observed (*P* = .07; Figure 3).

In the PPID‐only group, no difference was noted in baseline blood glucose concentrations (*P* = .93) or in post‐insulin blood glucose concentrations (*P* = .06) between combined and independent testing. Insulin administration had a significant effect on the 30‐minute postinjection blood glucose concentration (*P* = .01). No significant effect of combining the 2 tests was observed (*P* = .15; Figure 3).

In the ID and PPID group, no difference was noted in baseline blood glucose concentrations in the ID and PPID positive group (*P* = .56) or in post‐insulin blood glucose concentrations (*P* = .44) between combined and independent testing. Insulin had a significant effect (*P* = .01) on blood glucose concentrations, but no significant effect of combining the 2 tests was observed (*P* = .30; Figure 3).

**Figure 4:**
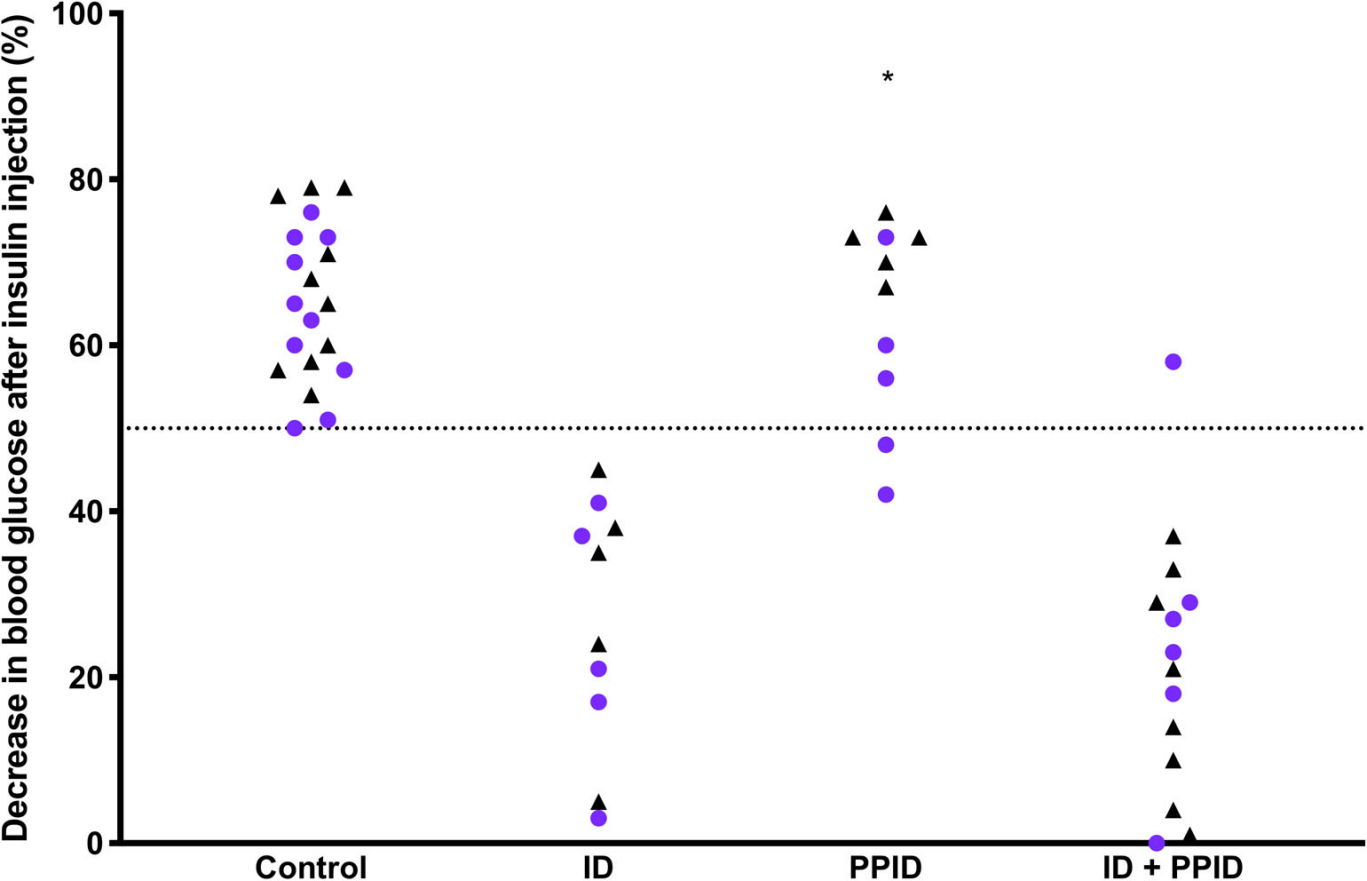
Percentage of decrease in blood glucose after intravenous injection of 0.1 mIU/kg of insulin in the 4 groups of horses. Black triangles represent data from independent testing and purple circles represent data from combined testing (* *P* < .05 between independent and combined testing)

No significant difference in the percentage decrease in blood glucose concentration was found in the control, ID‐only, or ID and PPID groups **(**Figure 4). In the PPID‐only group, a significant difference in the percentage decrease in blood glucose concentration was noted after insulin injection (*P* = .03; Figure 4) and 2 horses had a positive 2‐step insulin sensitivity test because neither reached the 50% threshold after 30 minutes after insulin injection with 48% and 42% decrease in blood glucose concentration in the 2 horses, respectively, with the combined testing. In the ID and PPID group, 1 horse had a negative 2‐step insulin sensitivity test and did reach the 50% decrease in blood glucose concentration 30 minutes after insulin administration, with a value of 58% with the combined testing.

### 3.3 Combined testing characteristics

Equivalence testing indicated that the observed differences in plasma ACTH and, blood glucose concentrations and percentage decrease in blood glucose concentrations between testing protocols were not significantly different from intra‐assay variability (*P* = .44, .21, and .34, respectively).

**Figure 5:**
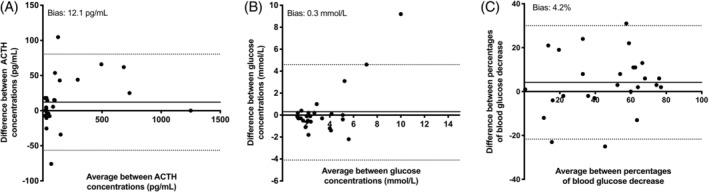
Bland‐Altman plot of the plasma ACTH concentrations after TRH stimulation tests performed as independent or combined testing (A), blood glucose concentrations performed as independent or combined testing (B), and percentage of decrease in blood glucose after intravenous injection of 0.1 mIU/kg of insulin performed as independent or combined testing (C**)**. The solid line represents the bias and the dotted lines represent the 95% limits of agreement. ACTH, adrenocorticotropic hormone

Bland‐Altman plots indicated that the mean bias for post‐stimulation plasma ACTH concentration with independent versus combined testing was 12.1 pg/mL (Figure 5A). The mean bias for blood glucose concentration post‐insulin administration with independent versus combined testing was 0.03 mmol/L (Figure 5B). The mean bias for decrease in the percentage of blood glucose concentration after insulin administration with independent versus combined testing was 4.2% (Figure 5c).

## 4 DISCUSSION

Our results show that a 2‐step insulin sensitivity test in combination with a TRH stimulation test is equivalent to the 2 tests performed independently, yielding results that are not significantly different from independent testing. In all 4 groups, no significant effect of combining the TRH stimulation test with the 2‐step insulin sensitivity test was observed on either plasma ACTH or blood glucose concentrations.

One horse in the control group tested positive for PPID during the combined testing. High plasma ACTH concentrations have been reported with stress, which could have contributed to the false‐positive result.^26^ All horses received the same housing and feeding conditions, thus it is unlikely that stress could have led to the increase in plasma ACTH concentration.^21^ Within‐horse as well as within‐laboratory variability should be considered for the unexpected increase in plasma ACTH concentration.^27^ Sample handling, analyte stability, assay variability, technical problems, or physiological variation all can contribute to inconsistent results. All samples in our study were submitted to the in‐house laboratory and centrifuged within 4 hours of collection for optimal results, limiting the effect of sample processing. Previous literature indicated that TRH stimulation tests should not be repeated within 24 hours in horses and found substantial interday variability for individual horses.^21^ The independent and combined testing were performed on separate days to ensure all blood glucose and plasma ACTH concentrations returned to preinjection concentrations, limiting the effect of the independent testing on the results of the combined testing.^21^ Because of the seasonal changes associated with plasma ACTH concentrations, all of the horses were tested during the early summer months, but it is possible that the false‐positive horse could have been transitioning either to PPID or to the autumnal active phase sooner than expected.^28^ Because no seasonal reference concentrations for the TRH stimulation test in horses have been established and variation in post‐stimulation plasma ACTH concentrations have been reported, it could not be concluded if the observed discrepancy was caused by the testing protocol or by factors related to the horse.^9^, ^29^, ^30^ Although further research is warranted to assess the repeatability of the TRH stimulation test during different seasons and in horses with and without PPID, a false‐positive result for PPID was not considered a major drawback because the current recommendation is to retest horses with unexpected results and vague clinical signs until classification is evident (Equine Endocrinology Group website, available at: http://sites.tufts.edu/equinendogroup, accessed January 10, 2019).

Two horses in the PPID‐positive group tested positive for ID during the combined testing and 1 horse in the ID and PPID‐positive group yielded a false‐negative result for ID during the combined testing. Although the changes were mild, they would have led to a misdiagnosis. A possible reason for the 2 false‐positive and 1 false‐negative ID cases in the respective PPID‐positive and ID and PPID‐positive groups could be interday variation associated with the 2‐step insulin sensitivity test. Although not specifically reported for the 2‐step insulin sensitivity test, considerable variation has been reported previously in the repeatability of insulin sensitivity dynamic testing.^31^, ^32^ Interestingly, those cases were mildly positive for PPID (diagnosed on the TRH stimulation test only and not on baseline). This finding could suggest that those horses could be metabolically unstable and their insulin sensitivity status could be changing. It has been shown that insulin sensitivity is not a fixed trait and that several factors such as fasting, age, and reproductive status can impact it.^20^, ^33^, ^34^

One of the limitations of our study was that hyperinsulinemia was not investigated as it would have allowed clearer determination of ID in those horses. Currently, the recommended test to diagnose hyperinsulinemia is the dynamic OST to assess the postprandial insulin response. In a previous study, TRH administration had a significant effect on insulin and glucose dynamics.^21^ This was not the case in our study as TRH injection showed no significant effect on blood glucose concentration (*P* = .24) suggesting that the discrepancy observed with the results of the 2‐step insulin sensitivity test when combined with the TRH stimulation test could be caused by intrinsic characteristics of the 2‐step insulin sensitivity test or the metabolic status of the horse rather than an effect of the combination. In addition, this combination of testing was developed to select and screen older and obese horses that are prone to develop endocrine disorders. When considering the possibility of overdiagnosing ID, it should be noted that clinically very little risk is associated with treatment such as dietary restrictions or foot care. Routine follow‐up testing allows veterinarians to identify false‐positive cases, which yields a better outcome compared to the high risk of not identifying and prophylactically treating horses at risk for laminitis before the first acute episode of the disease. Laminitis is a serious, debilitating disease for which there currently is no cure, and being overcautious with equivocal cases and educating owners about management would not be considered detrimental to the welfare and outcomes of these horses.

Another limitation of our study was the heterogeneity in the breeds and the small number of horses and ponies in each group. As it has been shown that there was a significant breed effect on insulin regulation, the low number of horses and ponies in each group combined with the range of breeds could have prevented us from detecting significant differences in plasma ACTH or blood glucose concentrations or percentage decrease in blood glucose concentration.^35^ As far as we are aware, although a breed effect has been clearly demonstrated, no breed specific diagnostic cut‐offs and reference intervals for insulin, glucose, or ACTH concentrations have been developed yet. Such data could improve interpretation of currently available endocrine testing protocols.

Based on our findings, the combined TRH stimulation test and 2‐step insulin sensitivity test is a very attractive diagnostic tool for equine practitioners in the field, allowing them to test a horse for both PPID and ID at the same time. Additionally, this diagnostic tool provides results for the 2 most common hormonal disorders in horses within 30 minutes. This combination also would be beneficial for owners because it would limit costs associated with repeated veterinary visits, requires no fasting before testing, increases the screening for both endocrine conditions, and initiating prophylactic laminitis treatment. We have shown that there is an increased rate of therapeutic failures when only 1 condition is addressed and the other is not recognized.^6^ This new testing modality allows earlier management of endocrine disorders and could decrease the rate of therapeutic failures observed when only 1 hormonal disorder is addressed.

## CONFLICT OF INTEREST DECLARATION

Authors declare no conflict of interest.

## OFF‐LABEL ANTIMICROBIAL DECLARATION

Authors declare no off‐label use of antimicrobials.

## INSTITUTIONAL ANIMAL CARE AND USE COMMITTEE (IACUC) OR OTHER APPROVAL DECLARATION

The University of Queensland Animal Ethics Committee approval number: SVS/392/17.

## HUMAN ETHICS APPROVAL DECLARATION

Authors declare human ethics approval was not needed for this study.
